# Digital Health Consumers on the Road to the Future

**DOI:** 10.2196/16359

**Published:** 2019-11-21

**Authors:** Rita Kukafka

**Affiliations:** 1 Department of Biomedical Informatics Columbia University New York, NY United States; 2 Department of Sociomedical Sciences Columbia University New York, NY United States

**Keywords:** digital health, health consumers, artificial intelligence, internet of things

## Abstract

Digital health is uniquely positioned to transform health care. This viewpoint explores the enormous benefits for health consumers when digital-first health care is embraced. Also, it explores what risks exist if surveillance capitalism takes over health care. Further, some solutions to prepare digital health citizens for the road ahead are also discussed.

## Introduction

Digital health is uniquely positioned to transform health care. Ubiquitous computing and the technological advancements of mobile-computing platforms and wearable consumer devices have enabled continuous monitoring of citizens and their everyday behaviors. This longitudinal data can be mined to disclose physiological and behavioral signatures of existing health impairments and may effectively provide predictive capabilities for health conditions yet to emerge. The most complete multi-modal data provide the best predictive capabilities, which then yield powerful pathways for preventing disease. Digital health consumers who provide the data have the most to gain, but they also have the potential for loss. This viewpoint explores the evolving lexicon and landscape of digital health, as well as some risks and benefits digital health consumers may face in the future. It also explores what risks exist if surveillance capitalism takes over health care. Finally, some solutions to prepare digital health citizens for the road ahead are discussed.

## An Evolving Lexicon of Terms for Digital Transformation 

Digital health is the broad umbrella term which, according to the World Health Organization (WHO), encompasses electronic health (eHealth) but has also been broadened to include connected software solutions (Internet of Things [IoT]), as well as computational methods applied to big data, genomics, and artificial intelligence (AI) [1]. Agreement on how to define related terms has been brewing for more than a decade (eHealth, medical informatics, telemedicine, and mobile health) [2-4], and a key benefit to proclaiming an umbrella term stems from the need to integrate related concepts that are agnostic to specificities, such as purpose and type of technology. Noteworthy, the *Journal of Medical Internet Research* (JMIR) advanced the need for a broad, unifying term decades ago [2], and early on embraced the ubiquitous and transformative nature of digital technologies:

e-health is an emerging field in the intersection of medical informatics, public health and business, referring to health services and information delivered or enhanced through the Internet and related technologies. In a broader sense, the term characterizes not only a technical development, but also a state-of-mind, a way of thinking, an attitude, and a commitment for networked, global thinking, to improve health care locally, regionally, and worldwide by using information and communication technology.

Regarding the expanding lexicon of terms, two points pertinent to the future path of digital consumers can be made. First, the quest to redefine terms will naturally occur as the field emerges, typically as a result of interdisciplinary, multidisciplinary, and transdisciplinary interactions of individuals and concepts in a way that transcends conventional field boundaries [5]. Nonetheless, it is the preservation of information contained in thousands of published articles and lessons learned that remain the core for understanding digital health consumers moving forward. The second, interrelated point also pertains to field boundaries and provides a clue to a future road. The WHO’s declaration to designate digital health as an umbrella term to encompass and broaden eHealth does not arise from expansion to new domains or of novel digital health technologies. Instead, it reflects the convergence of the eHealth landscape with fields such as AI, big data, and genomics. Big data, IoT, advances in computing power, and memory collectively set the stage for the future, enabling a remarkable unchartered path for digital health consumers and our conception of the space they encounter. It is at this intersection that the eHealth landscape is therefore positioned to advance and transform health care.

## Consumers’ Evolving Use of Digital Technologies

Just as the lexicon of terms has evolved to reflect expanding boundaries, so has the digital health consumer’s interactions with technologies also evolved. Consumer adoption of digital technologies has extensively increased [6], and it is also of note that consumers are evolving in their use of digital technologies [7]. However, as reflected in the preceding discussion, the most resounding change for digital consumers today is that they now reside amid a network of smart things that are capable of capturing their digital footprints. These smart things come with embedded sensors and wireless devices and form a state of extreme digital connectivity. The IoT first described by Kevin Ashton in 1999 describes this as a network of physical things that can sense other things using ubiquitous wireless connectivity and embedded sensors [8]. Ubiquitous and wireless connectivity is expected to grow in future years.

Today we live on a planet that has more smart things than human beings. The global IoT health care market, valued at US $5800 million in 2014, is expected to reach a value of approximately US $14,000 million by 2024 [9]. Around 29 billion connected devices are forecast by 2022, of which around 18 billion will be related to IoT [10]. The IoT streams in big data from smart things and can virtually and digitally connect inanimate and physical living things. With speed and efficiency, AI strategies are ideally adapted to managing and analyzing these continuous data streams in large amounts [11]. Unparalleled conveniences, benefits, and challenges persist at the intersection of IoT, AI, and health care [12]. If consumers are currently overwhelmed about how best to manage the growing number of smart objects at work and in everyday life, it is interesting to note that by 2030, each person will own 15 connected devices [13]. We are only experiencing the beginning of a fast-approaching deluge of smart things. 

## The Road Ahead for Digital Health Consumers

In academic literature, digital health interventions are commonly presented as ways to deepen patient activation or involvement in care, promote behavior change, and harness or promote self-management [14]. It was during the 1990s, as the internet exploded into public consciousness, that several eHealth terms began to emerge [2], and consumer use of the internet for health information reached exponential growth [15]. Both the emergence of Web 2.0 technologies and personal health applications, such as Google Health and Microsoft HealthVault, were viewed as having far-reaching consequences for patient involvement [16,17]. It was thought that health care would become democratized if patients had access to their medical records and access to the medical literature. Lay people would become more conversant with health and medical issues, and the traditional paternalistic paradigm was expected to shift. The IoT for digital health consumers may be the next evolution of the current internet into a network of interconnected smart things that not only gather information from the environment (sensing) and interact with the physical world, but also use existing internet standards to provide services for information transfer, analytics, application, and communication [18]. So how is the road forward expected to be different for digital consumers living in this world of ubiquitous and wireless connectivity?

In a highly connected, digitized world, more emphasis will be placed on the digital consumer. Rather than viewing the electronic health record (EHR) as the sanctified knowledge resource for each patient, data will be generated in diverse spaces and places (IoT), including the real world where patients work and live. There will likely be more information-sharing relationships: one-to-many, many-to-one, and many-to-any. This shift is also expected to improve the stickiness of smart devices, as providers get more value from data to monitor patients and as consumers become further incentivized to sustain use [7]. Smartwatches, smartphones, and an array of ubiquitous devices with embedded identification, sensing, and data exchange features enable data gathering in real-time for synchronous communications, personalized interventions, and monitoring patient processes and outcomes. Collectively, the IoT offers considerable potential for transforming health care from encounter-based care into connected continuous care [9]. These rapid changes that are interwoven into health care expand its capabilities and have only just begun to be documented and analyzed in the academic, critical, social-scientific literature.

Topol, in his book “Deep Medicine”, describes the virtual medical coach that is designed to support the needs of health consumers as the unfulfilled promise of big data, deep learning, and other AI tools [19]. One of the most important boons of deep medicine:

...is to empower not just physicians to be better at what they do, but to help all of us be as good as we can be at taking care of our own health.

The potential of the virtual medical coach would only be realized if it was merged with behavioral science methods to promote behavior change since so much of the burden of disease is related to poor lifestyle. As an example, IoT surveillance combined with individual sensors from smart devices and other relevant data might trigger behavioral nudges designed to address an impending asthma attack, rising blood pressure, increased BMI, or elevated glucose levels.

The inaugural editorial welcoming readers to JMIR, written by Gunther Eysenbach, highlighted the important role the internet plays in consumer empowerment and in redefining the traditional model of preventive medicine and health promotion. Since then, hundreds of JMIR’s papers have focused on participatory approaches to increase the health care experience for consumers and to empower them to take care of their health. The Journal of Participatory Medicine (JoPM), now published by JMIR Publications, pertains specifically to participatory medicine, a movement in which patients and health professionals actively collaborate and encourage one another as full partners in care. JMIR Publications, which is designed to explore the latest research in the field of digital health, provides a critical resource for the continued expansion of digital health technologies as the eHealth landscape continues to converge with the IoT, AI, big data, and genomics.

## Risks and Benefits of Electronic Health for Digital Health Consumers

There have been some promising examples of eHealth applications coupled with AI and the IoT. In the area of health monitoring and risk prediction, AI can use raw data from sensors and machine-learning algorithms can then be trained to recognize patterns from the raw data inputs. Patterns can then be categorized as indicators of an individual’s behavior and health status, which allow patients to understand and manage their health as well as share data with medical providers. For example, a recent literature review published in JMIR analyzed the literature from 2010 to 2018 and yielded 1849 pertinent articles that combined AI with the latest technologies for diabetes management and decision support [20]. Ultimately, 141 papers were included in the review, which demonstrated the potential of AI to enable diabetes solutions in the context of multiple critical management issues, including blood glucose prediction and control, detection of adverse glycemic events and risk, and patient personalization. In the area of precision medicine, eHealth combined with IoT and AI can be used not only to predict outcomes but also to predict future patients’ probability of having diseases, including the probability of not having a disease [21].

As IoT and AI in health care converge to be transformative for the digitally engaged consumer, there are also new risks and challenges. Data for its own sake does not have much value. It is only when information and knowledge are derived from data that actionable utility becomes possible, however, the end-users of this data may vary enormously. This begs the question: what happens when the “underlying model of improved health care and consumer empowerment” becomes “control of humans for profit?” Zuboff raises concerns in her book “The Age of Surveillance Capitalism” about what she calls behavioral futures and prediction products [22]. According to Zuboff, surveillance capitalism: unilaterally claims human experience as free raw material for translation into behavioral data. Although some of these data are applied to service improvement, the rest are declared as a proprietary behavioral surplus, fed into advanced manufacturing processes known as “machine intelligence,” and fabricated into prediction products that anticipate what you will do now, soon and later.

Digital health consumers may stand more to lose than their privacy. What is at stake is their autonomy and right to make their own decisions about their health. Nudging health consumers based on AI behavioral predictions may seem praiseworthy when the end-user goal is to improve health, but what happens when consumers are nudged solely to purchase specific products, seek a specific treatment, or vote on a specific piece of health care legislation? From a health care viewpoint, this begs the question of how consumer advocacy groups could take control of digital health from corporate and political interests so that benefits could be gained without the harms that could result from an algorithmic technocracy concerned with profit and control.

Indeed, there are many other challenges digital health consumers will face on their road to the future. Digital consumers may be comfortable relying on AI when using Uber and Amazon, but that is different than relying on AI to guide their medical treatment plans. This concern is justifiable considering that, of the millions of digital health devices available for consumers today, only a small fraction have been tested and the evidence for those that had been evaluated was of low quality [23,24]. Smart and not so smart devices may generate diagnoses, make recommendations, and in some instances, may be authorized to act [25]. Current Health Insurance Portability and Accountability Act (HIPAA) regulations, specifically the Security Rule, discuss the accessibility, integrity, and confidentially of all electronic, protected, health information, but they do not specifically govern IoT devices. Many unanswered questions highlight the need for additional legislation dictating who is responsible for the protection of IoT, such as, does an app or IoT device manufacturer owe consumers a HIPPA level of security for maintaining records of health insights? It is expected that the IoT industry may face additional regulations by the Food and Drug Administration, the US Department of Health and Human Services Office of Civil Rights, and other entities, plus health care–specific regulations.

Calls to action have been effective at spreading the message that digital health will bring patient empowerment, while others have been encouraging a more balanced view that seeks to capitalize on the benefits of digital health while minimizing the risks of potential harms [26-28]. 

## Conclusions

We have only just begun to scrape at the ethical implications for future digital health consumers. It is often challenging to assess who the main drivers of this “creative destruction” are [29]. There are so many stakeholders, most of them from nonhealth conglomerates, and many of them transnational. The speed of development is surprisingly rapid, and since we are part of the change it is hard to step back to see precisely where we are going. Predicting human behavior to empower digital health consumers to prevent disease may not be as profitable as trading behavioral predictions for profit. Those of us working in academia at the intersection of digital health, ethics, and social justice must be aware and stay ahead of the challenges digital health consumers will face in the future. The road digital health consumers take into the future may be very different from roads taken in the past.

## Abbreviations

AI: artificial intelligence
eHealth: electronic health
EHR: electronic health record
HIPAA: Health Insurance Portability and Accountability Act
IoT: Internet of Things
JMIR: Journal of Medical Internet Research
JoPM: Journal of Participatory Medicine
WHO: World Health Organization
